# Puberty health intervention to improve menstrual health and school attendance among adolescent girls in The Gambia: study methodology of a cluster-randomised controlled trial in rural Gambia (MEGAMBO TRIAL)

**DOI:** 10.1186/s12982-022-00114-x

**Published:** 2022-07-16

**Authors:** Vishna Shah, Penelope Phillips-Howard, Julie Hennegan, Sue Cavill, Bakary Sonko, Edrisa Sinjanka, Nyima Camara Trawally, Abdou Kanteh, Francois Mendy, Amadou B. Bah, Momodou Saar, Ian Ross, Wolf Schmidt, Belen Torondel

**Affiliations:** 1 Environmental Health Group, Department of Infectious Diseases, London School of Hygiene and Tropical Medicine, Keppel Street, London, WC1E 7HT UK; 2 Department of Clinical Sciences, Liverpool School of Tropical Medicine, Pembroke Place, Liverpool, L3 5QA UK; 3 Maternal, Child and Adolescent Health Program, Burnet Institute, Melbourne, Australia; 4 Freelancer, London, UK; 5 Nutrition Theme, MRCG Keneba, Medical Research Council Unit, Banjul, The Gambia; 6 Nova Scotia Gambia Association (NSGA), Banjul, The Gambia

**Keywords:** Menstruation, Menstrual health, Menstrual hygiene, Menstrual health and hygiene, Intervention, School attendance, Adolescent girls

## Abstract

**Background:**

Menstrual health (MH) is a recognised global public health challenge. Poor MH may lead to absence from school and work, and adverse health outcomes. However, reviews suggest a lack of rigorous evidence for the effectiveness of MH interventions on health and education outcomes. The objective of this paper is to describe the methods used in a cluster-randomised controlled trial to estimate the effect of a multi-component intervention to improve MH and school attendance in The Gambia.

**Methods:**

The design ensured half the schools (25) were randomised to receive the intervention which comprised of the following components: (i) Peer education camps and menstrual hygiene laboratories in schools, (ii) Mother’s outreach sessions, (iii) Community meetings, and (iv) minor improvements of school Water Sanitation and Hygiene (WASH) facilities and maintenance. The intervention was run over a three-month period, and the evaluation was conducted at least three months after the last intervention activity was completed in the school or community. The other 25 schools acted as controls. Of these 25 control schools one Arabic school dropped out due to COVID-19. The primary outcome was the prevalence of girls missing at least one day of school during their last period. Secondary outcomes included: Urinary Tract Infection (UTI) symptoms, biochemical markers of UTI in urine, Reproductive Tract Infection symptoms, self-reported menstruation related wellbeing, social support and knowledge, perceptions and practices towards menstruation and MH in target school girls. In addition, a process evaluation using observations, routine monitoring data, survey data and interviews was undertaken to assess dose and reach (quantitative data) and assess acceptability, fidelity, context and possible mechanisms of impact (qualitative data). Cost and cost-effectiveness of the intervention package will also be assessed.

**Conclusion:**

Results will add to scarce resources available on effectiveness of MH interventions on school attendance. A positive result may encourage policy makers to increase their commitment to improve operation and maintenance of school WASH facilities and include more information on menstruation into the curriculum and help in the reporting and management of infections related to adolescent menstruation.

*Trial Registration* PACTR, PACTR201809769868245, Registered 14th August 2018, https://pactr.samrc.ac.za/TrialDisplay.aspx?TrialID=3539

**Supplementary Information:**

The online version contains supplementary material available at 10.1186/s12982-022-00114-x.

## Introduction

Menstruation is a normal and natural monthly occurrence; however, it is often associated with negative connotations [1]. Globally, menstruators around the world have developed their own personal strategies to cope with menstruation, which vary greatly from country to country, individual’s personal preferences, available resources, local traditions, cultural beliefs and knowledge or education level. Menstrual health (MH) is a state of complete physical, mental, and social well-being and not merely the absence of disease or infirmity, in relation to the menstrual cycle. It requires information about the menstrual cycle and self-care; materials, facilities and services to care for the body during menstruation; diagnosis, care, and treatment for menstrual discomforts and disorders; a positive and respectful environment which minimises psychological distress; and freedom to participate in all spheres of life [2]. Challenges associated with effective MH include lack of access to clean, effective absorbents; inadequate sanitation facilities to change, clean and dispose of absorbents; lack of access to soap and water; lack of privacy; inadequate social support; lack of knowledge; and presence of taboos and restrictions [3–7]. All these challenges can lead to absence or distraction at school or work, adverse health outcomes such as urinary tract infections (UTI) or reproductive tract infections (RTIs) or psychosocial consequences of menstruation including depression, anxiety, shame, fear [3–7]. A limited body of evidence supports the premise that two types of RTIs, bacterial vaginosis [8–10] and vulvo-vaginal candidiasis [11] can be associated with different unhygienic menstrual hygiene management practices.

Poor MH may lead to school absenteeism, disengagement or withdrawal from school, affecting a girls’ ability to pursue higher levels of education [11–14]. However, the quantity and quality of evidence is sparse, due to limited studies, small size of studies, and challenges assessing attendance outcomes (challenges of accuracy of school registers and self-reporting biases) [15–19].

Staying in school has shown to protect girls against early marriage, teen pregnancy, and HIV infection, with school-girls reporting less frequent sex, and fewer partners with less age disparity [20–22]. Therefore absence from school has been of particular interest to international development organisations and research bodies, and resulted in a growing awareness of the need to improve MH in school-girls for global health and development [3, 23, 24]. Although, reviews suggest a lack of rigorous evidence for the effectiveness of MH interventions on school attendance [11, 12].

The Gambian government has recognised the need for improving MH among adolescent school girls and has an initiative to supply free disposable sanitary pads to all public schools [25], rigorous evaluation of this initiative is yet to be done.

A study conducted in The Gambia (MRC-PHIND) among school girls in the Kiang region suggested challenges related to MH at home or school could be associated with negative health outcomes and school absenteeism [26, 27]. The study showed that students (boys and girls) had poor knowledge about menstruation, largely due to the need for secrecy and cultural beliefs [26, 27]. Findings from a mixed methods approach suggested that lack of knowledge of menstruation and adequate absorbent materials led to embarrassment and fear of teasing, and this, together with pain and poor school water sanitation and hygiene (WASH) facilities contributed to school absenteeism. It also highlighted an unmet need for engaging girls, boys and parents, to enable girls to better manage both the emotional aspects of menstruation (depression, embarrassment, anxiety and distress) and the physical aspects (management and use of appropriate materials to eliminate leakage of menstrual blood and hygienic habits practices) [26].

Formative research cross-sectional studies conducted in The Gambia between November 2015 and December 2017 [28], showed 27% of school girls reported missing at least one day of school during their most recent period. A third of all participants reported they would miss school when menstruating because of pain, 15% reported fear of staining was a deterrent to attending school, and 62% reported not feeling comfortable when using sanitation facilities at school when menstruating.

The objective of this paper is to describe the methods used in a cluster-randomised controlled trial to estimate the effect of a multi-component intervention to improve MH and school attendance in The Gambia.

## Methods

### Study aims and objectives

This trial aimed to evaluate the effectiveness of the multicomponent intervention in improving MH and school attendance among target school girls in rural Gambia. The specific objectives were to:

1. Assess the effect of the intervention on menstruation-related school absence during the last period (primary objective) among target school girls.
2. Assess the effect of the intervention on reported UTI and RTI symptoms, and biochemical Markers of UTI in urine (nitrites and leucocyte) among target school girls.
3. Assess the effect of the intervention on menstruation related wellbeing and social support among target school girls.
4. Assess the effect of the intervention on improving menstrual management practices among target school girls.
5. Assess the effect of the intervention on improving menstrual needs including reproductive health knowledge and attitudes towards menstrual taboos, among target school girls.
6. Assess the cost and cost-effectiveness of the intervention.
7. Assess acceptability, coverage, uptake and fidelity of the intervention package.

### Study setting

The Gambia is the smallest country in mainland Africa, with the width of the country never exceeding 50 km [29], and is predominantly Muslim (96%) [30]. In 2015, 49% of the population were living below the national poverty lines [31]. Almost 60% of the population is under 25 years [30]. The 2019/2020 Demographic Health Survey found literacy (can read aloud) rates to be higher among males than females at all age groups. The literacy rate for the population between 15 and 24 years among males was 71% and for their female counterparts was 63%. At the 45–49 age group the difference was even larger; males (64%) and female (23%) [32]. The survey also showed the net attendance ratio for primary school is 74% and drastically drops to 46% for secondary school. There is a large variation in secondary school net attendance ratio between urban (51%) and rural (33%) areas [32]. Schools in The Gambia are broadly classified as English-based or Arabic-based schools. The English-based schools are free public schools, whereas Arabic schools are private and mainly focus on Quranic education [33]. English-based schools receive an annual free supply of disposable sanitary pads from the Ministry of Basic and Secondary Education (MoBSE) [25], the schools then decide how to distribute this supply to the girls in the school. However, the Arabic schools do not get such a supply from the government. The education system is classified into Lowe Basic School (LBS) (Grade1-6); Basic Cycle School (BCS) (ECD-Grade 9); Upper Basic School (UBS) (Grade 7–9) Senior Secondary School (SSS) (Grade 10–12) [33]. Many schools may exist as double shift schools, where certain grades attending in the morning and others in the afternoon [34]. The national curriculum contains no teaching specifically on menstruation, though students typically receive some basic information on male and female anatomy in the science and population and family life classes.

**Fig. 1:**
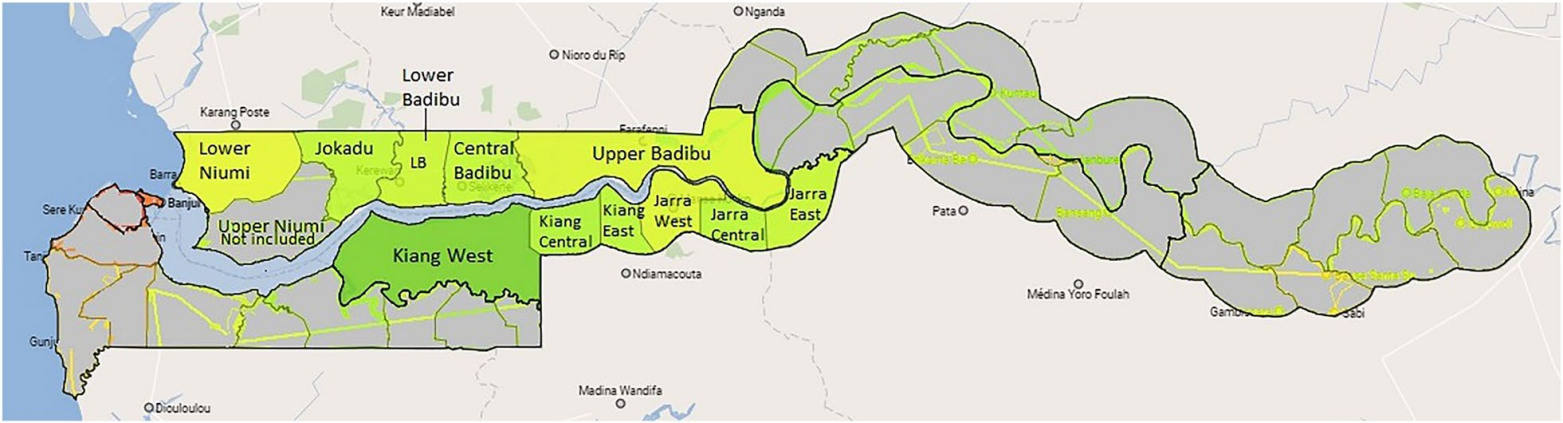
Map of The Gambia. The green areas on the map highlight the regions the study was conducted in. Adapted from: https://www.citypopulation.de/en/gambia/admin/ [36]

The study was conducted in 50 schools across two regions of The Gambia; the Lower River Region (LRR) (Kiang West, Kiang Central, Kiang East, Jarra West, Jarra Central and Jarra East Districts) and North Bank Region (NBR) (Lower Badibou, Central Badibou, Upper Badibou, Jokadou and Lower Niumi Districts) (Fig. 1). These regions are predominantly rural, and agricultural, with most people engaging in production of groundnuts, millet and rice [35].

### Study design

This study used a cluster randomised control trial design, with two arms, one arm received the MH intervention package (intervention arm) and the other arm did not receive any intervention (control arm). The intervention was delivered at the beginning of the school year, from October-December 2019 and three months later (February 2020) end-line outcome data collection started. Due to COVID-19 trial procedures had to be stopped on 19th March 2020 after completion of data collection in 44 schools. Data for the remaining schools was collected in November–December 2020.

Since the intervention was implemented in schools and communities from the village where the school is placed, the village was chosen as the unit of randomisation, with one school selected per village for the study.

### Eligibility criteria for villages and schools

All the villages from LRR and NBR districts with at least one Arabic school, or BCS or UBS or SSS were listed. From the list, 50 villages were randomly selected. One school per village was enrolled. If a village has two or more schools, the school with the largest number of enrolled girls was selected, to ensure sample size was reached. In total, there were 24 schools from the LRR region and 26 from the NBR region. With a total of 14 Arabic-based schools and 36 English-based schools. However, one Arabic-based school from the control arm dropped out due to COVID-19.

### Eligibility criteria for participants

For the end line data collection, post menarche girls over 13 years old were enrolled. Only girls that could provide informed consent or assent were enrolled, therefore, participants with mental disabilities were excluded. Physical disability was not an exclusion criterion as MH may be of particular interest in these groups.

The study population reflects the target population. The vast majority of eligible girls in each school were enrolled for outcome assessment. In schools with more than 300 eligible girls, the number of enrolled girls was capped at 300 as it was statistically inefficient to recruit more than that. In schools with fewer than 300 eligible girls, all eligible girls were selected. In schools with more than 300 eligible girls, a representative sample from each grade, excluding exam grades (Grade 9 and 12) were selected, if the eligible girls in the non-exam grades were not enough to reach the 300 cap, then girls from the exam grades were approached till the cap was reached. Girls in exam grades had busy schedules with many extra classes and limited time, therefore we opted to use non-exam grades first. Previous studies [26–28] found there was no significant difference in opinions if the exam grades were excluded, as long as there was representation from the other grades. If girls from Grade 9 and 12 were selected, they were approached at a time advised by the teachers and students, in most cases this was after school hours.

### Informed consent

Consenting for minors (under 18 years) was done by inviting a parent or guardian to the school, where the study was explained and the information sheet read to them, and it was reiterated to each student that they could withdraw at any time without giving a reason and without having any repercussions. They were then given an opportunity to ask any questions they may have. After which if they accepted, they were asked to sign or put their thumb print on the consent form. An independent witness was present for the consenting discussions. Prior to the survey, participants went through the same process. After agreeing to participate in the study, participants were asked to sign a consent form (Additional file 1: Appendix S1), minors signed the same form their parent or guardian had signed (Additional file 2: Appendix S2).

### Intervention development

A stakeholder workshop (with parents, teachers, students, clinicians, regional education officers and NGOs), held in May 2017, in Keneba, The Gambia, discussed initial findings [26, 27] from the formative research on MH in The Gambia, the problems and possible solutions. A four component intervention package was created, that could be included in existing programmes in these communities: (i) Peer Education camps, which would involve girls and boys in schools, discussing puberty and menstrual management issues; (ii) Mother’s outreach sessions to discuss issues linked to puberty, menstruation, menstrual absorbent and hygienic practices with mothers; (iii) Community meetings around puberty and menstruation, to involve the men in the communities; and (iv) minor improvements in school WASH infrastructure.

This intervention package was initially tested in two schools within the West Kiang Region, feedback from this testing was used to refine the material further. After which in 2019, a pilot intervention trial was run to further refine the material, test feasibility and acceptability of components of the intervention, test data collection tools, train implementers and enumerators and inform sample size calculation decisions for the main trial. The pilot was conducted in three intervention schools and three control school, with a total sample size of n = 299. Intervention schools used in the pilot were excluded from the main trial.

### Description of the intervention

#### Peer education camps and menstrual hygiene laboratories

Our previous study [26] showed that students gained relatively little knowledge about puberty and menstruation from their teachers and they felt more comfortable to talk with their peers. To address this lack of knowledge, Nova Scotia Gambia Association (NSGA) was subcontracted as a partner, to deliver an innovative two-session MH training per school. NSGA is a Canadian NGO working with adolescents and communities in The Gambia since 1985, it is known for using an interactive and direct engagement approach, dealing with different public health issues (Malaria, HIV, early marriage). The NSGA team went through a four-day training before the pilot intervention and again before the main trial intervention. The training covered aims of the intervention and familiarisation with intervention content.

In preparation for the first session, the school teachers were asked to select 15 boys and 15 girls that were outspoken and respected by other students. The first session was a full day session delivered to the 30 students, it included an introduction to the study, discussion about puberty education affecting both boys and girls, and discussions about menstruation. These 30 students served as menstrual ambassadors, to share information about puberty and MH practices with other students. The session had role-play activities, that showed the students how they can talk to others students about what they have learnt, they were also encouraged to talk to adolescents outside of the school setting. They were given different options of how they could spread the message such as through school assemblies, use of peer educator rooms in school etc. It was explained to the ambassadors that it was their responsibility to share what they learnt in the sessions with those that could not attend. This is the ethos of NSGA and the students are used to this approach. At the second session (the following day), all the school girls aged 13 years and above, were invited to attend a menstrual hygiene laboratory (if the school was large, this ran as multiple sessions, to ensure the group size was manageable). During this session, girls were split up into similar age groups, so as to ensure they were more comfortable discussing and asking questions. They were taught more in detail about menstruation, menstrual management practices, how to prepare for the period, tracking the menstrual cycle, information about absorbent materials and pain management (e.g., exercise, applying heat and or pressure, stretching, hydration, medical-herbal and pharmaceutical). The 15 ambassador girls that attended the first session also attended the menstrual hygiene laboratory session, and supported the NSGA staff in facilitation. One poster was given to each school after the training, with information about menstrual hygiene practices, physiological knowledge and tips for managing pain and how to prepare for your cycle. The same information was also included into a leaflet which was given to each girl that attended the training. The sessions with the students were full day sessions, with many icebreakers and activities throughout to keep the students engaged. Sessions were carried out on weekends, to reduce impact on school learning.

#### Mother’s outreach

Our previous study showed that mothers were one of the first point of contact for advice about how to manage menstruation for girls [26], however, mothers have limited knowledge about menstruation and also have different perceptions about how menstruation should be managed. At the stakeholder meetings, it was suggested that parents should be involved in the intervention package to be able to support girls with good information and a more open approach. Therefore, we decided to organize mothers’ meetings in each school. The school admin/teachers were asked to identify 20 mothers that were active, confident, vocal and easy for others to approach. These mothers were invited for a two-day meeting. The meeting ran from 9 am to 12/1 pm, we opted to have half day meetings, as we found concentration levels were low in the afternoon, and mothers were rushing to get back to other chores. During the first session, issues about importance of girls finishing school, and information about puberty and reproductive issues were discussed. The second session discussed menstruation in greater detail, role-play approaches of talking with children about menstruation, and menstrual hygiene management (including strategies for using different type of absorbents, absorbent and body hygiene practices, managing pain). Mothers were shown all the types of absorbents and explained their advantages and disadvantages and how to use them. Mothers were encouraged to work with the school administration to help maintain the WASH facilities, examples of assisting with cleaning school facilities or making soap for the school were given. The mothers were also encouraged to share this information with other women and adolescents in the community to increase discussion and awareness about menstruation. During the sessions, the mothers practiced different interactive methods of sharing the information. The sessions were conducted by two female facilitators (one nurse and one traditional influencer) who have experience discussing sensitive topics and have an energetic and engaging approach. The facilitators had three days training to discuss content of training materials and how they could engage the mothers, after which they had a practice session with a group of mothers that were not part of the study, followed by another training session to go over feedback and address any challenges faced.

#### Community meetings

Following suggestions from the stakeholder workshop to involve men in discussions about puberty, menstruation and the importance of girls schooling, a community meeting in the evening after prayers was considered to be the best opportunity to discuss these topics. One community meeting was conducted in each community, where both men and women attended. The sessions discussed the above mentioned topics and provided an opportunity for community members to ask questions. The sessions were arranged through the village elders, village development community chairman, and a religious elder, to increase acceptance of the program and promote attendance. The meeting lasted an hour or two depending on the level of engagement from the attendees. The sessions were conducted by two facilitators (one female and one male), who have an energetic approach and are used to discussing sensitive topics within communities and are respected by the communities. The facilitators had a day training to go over context of the session, after which they had a practice session in a community that was not part of the study, followed by another training session to go over feedback and address and challenges faced.

#### Improving school WASH facilities and maintenance

During the stakeholder meeting, representatives from all the schools showed willingness to help with the suggested interventions, and WASH improvements were seen as a major intervention priority. In the intervention schools, the school management team, mother’s outreach group and peer education camps were engaged to implement inexpensive measures including ensuring water storage containers were close to the toilets, and hand-washing stations were present with kettles (water pouring device commonly used in The Gambia), water and soap. After an initial WASH spot check (Additional file 3: Appendix S3), the team discussed with the school management team, simple changes that could be made to the current system where required, e.g., separating toilet facilities for boys and girls where possible, creating simple doors to increase privacy etc. A tailored TOR was created with each school at the beginning of the study with the agreed maintenance tasks. The agreement also included that the study team would provide materials to improve WASH facilities in a step wise manner. First the schools were provided with 100 L water tanks to be placed near the toilets and ensure they were always filled together with two handwashing stations and four kettles. After which at the next visit, they were provided with soap and materials to make tippy taps, which should also be placed near the toilets.

Initially part of the WASH intervention included an aspect of absorbent disposal facility in the schools, as most students dispose their used disposable pads into the pit latrine and the study team recognised that this would cause the pit latrines to filling up quickly. However, after further discussions with the girls and mothers, it was realised there were many differing views on used material disposal, some felt the material should be burnt, while others felt burning would lead to infertility and therefore was not an option. Because an intervention provided by the researchers could not thus be standardised across schools, this aspect was removed. This was replaced by including discussion on disposal options in the outreach sessions, where participants were encouraged to use the method they were most comfortable with, instead of throwing the material into the latrine. During the formative research no girl reported methods of disposal as a barrier to attending school.

All intervention components were simultaneously run in all intervention schools over the intervention period.

### Randomisation, allocation and blinding

Villages/schools were randomised within 10 strata, with one stratum with 2 schools, 4 strata with 4 schools, a further 4 strata with 6 schools, and one stratum with 8 schools. Strata were formed by a combination of: (1) region (Lower River vs North Bank), (2) English vs Arabic schools, and (3) level of socio-economic development. These factors were a priori assumed to be associated with menstruation-related school absence and the effect of the intervention on this outcome. The level of socio-economic development was done in two ways: (1) the research team created a level of urbanisation scoresheet and visited all the villages to complete the score, to determine level of development; and (2) Six study staff familiar with the study area were asked to rank the study villages according to the perceived level of development. Ranking from both methods was compared and any ranks that varied were discussed and additional information about the villages was sought or discussed with two other staff in the demographic health survey department. The ranks were collapsed into three equally sized categories of development (high, middle and low).

Randomisation of villages/schools within strata was further restricted by excluding allocations that did not meet pre-specified balance criteria, following methods described by Sismanidis et al. [37]. The variables used for restriction were: access to a tar road (binary), availability of electricity (binary), availability of mobile phone tower (binary), predominant building material of houses, i.e., mud vs other (binary), total number of girls in enrolled school, number of girls of menstruating age in enrolled school, distance to nearest major town (km), number of standpipes in village per number of household compounds, percentage of houses that own a TV dish. For binary variables, randomisations were accepted where arms differed by no more than 5% points. For continuous variables, randomisations were accepted in which the relative difference in means between arms did not exceed 20%, except for the school level variables “total number of girls” and “total number of girls of menstruating age” for which a 10% relative difference was accepted. Randomisations were done 50,000 times, with 20 allocations meeting the criteria. The number of possible allocations was about 1.2 * 10^7^out of 2.9 * 10^10^ possible allocations—a restriction factor of 99.96%. Of the 20 acceptable randomisations, we chose one at random as final allocation. The validity of the allocation was checked in 281 allocation meetings the balance criteria. Each pair of clusters was assigned together to the same treatment arm with an average of 138 allocations, ranging from 51 to 210 of the 281 allowable allocations (disregarding the stratum with only two villages that could not be allocated to the same treatment).

The trial manager was aware of the allocations for logistic purposes, however the enumerators collecting the end line survey data, and the statistician analysing the data were kept blinded to the allocations. After initial statistical analysis, the allocations would be revealed to the statistician to allow for subgroup analysis.

After randomisation, one village was removed from the study as due to miscommunication, the actual number of eligible girls in the selected school (Arabic) was far lower than reported earlier. This village was replaced by the closest village within the same region with an Arabic school.

A further school dropped out on 17th March after randomisation, just before data collection due to concerns regarding COVID-19 and maintained their refusal status even after data collection was resumed in November.

### Sample size

The 2019 pilot data showed a slightly lower prevalence of the primary outcome (Proportion of girls with at least one-day absence during last menstrual period), than previous studies, at 16.8%. We assumed that the intervention would lead to a 33% reduction in the primary outcome, which resulted in a crude sample size (ignoring clustering) of 637 girls per study arm. In the pilot study, the ICC for the primary outcome was 0.026. The number of girls of menstruating age per enrolled school (i.e., cluster size) was 75 girls, resulting in a design effect of 2.9 and an overall sample size of 1862 girls per arm, or 25 schools per arm. An average of 75 menstruating girls per school was used as an estimate to calculate how many schools needed to be enrolled into the study, However, we identified that some schools had less than 75 menstruating girls, to account for this, we allowed to select all the menstruating girls in the school, so that larger schools could compensate for smaller schools while ensuring that the sample size was reached. Although we did introduce a maximum cap of 300 menstruating girls per school, as any more than this would be statistically inefficient and not reduce the number of schools that needed to be visited.

### Process evaluation

A detailed mixed-methods process evaluation based on the MRC Process Evaluation Framework [38] was conducted to explore three core functions of the intervention: implementation, mechanisms of impact and context, to provide guidance for sustainable and scalable implementation of the intervention should it prove successful. We collected data on information on fidelity, reach, acceptability, context and mechanism of impact using different tools (Table 1): (i) end-line quantitative survey to assess reach and dose; (ii) interviews with facilitators within a week of completion of intervention delivery to assess feasibility, fidelity (challenges to delivery, barriers to uptake, how the intervention was delivered, assess if implementation varied from what was planned and reasons for variation) and contextual factors (logistical factors impeding or facilitating, engagement level of participants, unexpected participants, adaptations required and factors for these); (iii) in-depth interviews (IDI) with 19 girls, 13 boys and 6 teachers, and focus group discussions (FGDs); 6 with girls and 6 with mothers, conducted after end-line collection to assess acceptability, uptake, and possible mechanisms of impact; (iv) data from three unannounced visits checking on WASH facilities throughout the study to assess changes in WASH over time, and assess if schools were making and maintaining changes discussed, if not, to understand barriers to implementing and maintaining changes; (v) routine data collected as part of the implementation (reports, pictures, registration forms from implementers; school visitation logs); and (vi) unannounced observations of facilitators to ensure schedules were followed and assess quality of intervention delivery and report on context factors (logistic factors, engagement level of participants).

**Table 1:** Summary of process evaluation

Evaluation measures	Research questions	Data collection methods/sources	When was data collected
RA1.1: implementation			
Fidelity	How did: (a) training and (b) actual implementation of each component of the intervention vary from what was planned? Schools were visited as scheduled What were the barriers and facilitators to implementation fidelity? What adaptations were made?	Unannounced observations In-depth interviews (IDIs) with implementers WASH changes—interviews with teachers in charge of implementation	Observations: over the course of implementation Interviews: within a week of completion of intervention delivery WASH changes—at each spotcheck
Coverage and uptake	What proportion of adolescents participated in the intervention? What proportion of mothers participated in the intervention? What proportion of community members participated in the intervention? Was there any contamination?	Unannounced observations Pictures and videos of training Attendance registers collected by implementers In-depth interviews (IDIs) with implementers End line questionnaire survey	Observations and pictures: over the course of implementation Attendance registers and IDI—after completion of intervention delivery End line survey done months after intervention delivery
RA1.2: mechanism of impact			
Responses to and interaction with the intervention	Which components of the intervention were best accepted and adopted by adolescents, mothers and community members? What were barriers to acceptability and uptake?	Unannounced observations Focus Group Discussions (FGDs) and IDIs with adolescents (boys and girls), mothers, community members and teachers WASH observations and discussions with teachers	Observations and discussion with teachers about WASH changes: over the course of implementation FGDs and IDIs after the end-line survey
Interactions and consequences	How did components of the intervention interact? Were there any unanticipated pathways or consequences?		
RA1.3: context			
Proximal and distal	What social, cultural, political, and logistical factors impede or facilitate (or were affected by) how the intervention was implemented, and how adolescents, mothers, community members and were able to engage with and adopt it What were contextual reasons for adaptations to the intervention and its delivery?	Stakeholder Interviews FGDs, IDIs with adolescents, mothers, community members and teachers Unannounced observations Reports from various sources Engage with ministry of education to keep track of programs they ran and other potential programs	Continuous—throughout the course of the study

### Theory of change

The theory of change (Additional file 4: Appendix S4) was based on the Behaviour Centred Design (BCD) framework developed by Aunger and Curtis [39]. It shows the barriers to school attendance that were identified during formative research in The Gambia [26–28]. The barriers related to environmental, social and physiological factors, exploring these barriers resulted in ensuring the intervention package addressed all the barrier components identified. Addressing environmental barriers would ensure that girls had access to the hardware that they needed to maintain good MH resulting in an increase chance of attending school while menstruating. Improving social factors would result in more social support, improved communication about menstruation, a place for girls to ask questions about what they were experiencing, reduced teasing creating an overall positive attitude towards menstruation and making girls more comfortable attending school while menstruating. Addressing the physiological barriers such as pain and discomfort and low confidence through the various pathways would increase the ability to walk to school, and concentrate while in school.

### Outcome measurement

All outcomes, apart from biomedical markers of UTIs, were measured at end-line through an enumerator-administered survey. After completion of the survey, participants were asked to provide a midstream urine sample to measure biomedical markers of UTIs, through dipstick analysis. All survey questions (Additional file 5: Appendix S5) were translated into the local languages (Mandinka, Wollof and Fula), and back-translated to confirm wording. School girls’ surveys were verbally administered by trained female enumerators. Enumerators received 10 days of training, initially they were trained on the purpose of each question, and ways to ask the questions, after which they had a chance to trial asking the questionnaire to girls from schools that were not part of the study, and further training was conducted to address any challenges that may have been faced during the trial. Data from school girls was collected in a private room on the school grounds. As menstruation is a sensitive topic, female enumerators were selected to facilitate participation in the study. Survey responses were entered on tablets using REDCap software version 8.9.2 [40].

### Primary outcome

#### School attendance

The primary outcome for this study was the proportion of girls with at least one-day absence during their last period. We aimed to collect school register data as a gold standard method to measure school attendance and to be able to compare and validate the survey data used to measure the primary outcome. However, we found lots of inaccuracies in register data, and data could not be collected from all the schools. Previous studies have also found registers to be inaccurate, irregularly completed, and difficult to use due to teachers not completing daily, and girls using different names for school enrolment and study enrolment [17, 41, 42]. Thus, we will perform a comparison exercise only with the data from the available schools which have the most complete data to be able to estimate validity of the survey data.

The MENISCUS study in Uganda [15] had success in using self-completed diaries to collect information about school attendance, however formative research in The Gambia found this to be an unsuitable tool for the primary outcome as the diaries are often left empty. This difference could have been due to differences in literacy level, or not being able to have the diaries in the local language, since the local languages are not written, or students in The Gambia had never done such activities. Therefore, for this context, we decided to use self-reported school absence during the last period as the measure to estimate the primary outcome. The question that we used in our questionnaire to measure this outcome was: In the last 30 days how many days of school did you miss because of menstruation? Girls had the option to say they did not menstruate in the last 30 days, in this case, a further question was asked: During your last period, how many days of school did you miss because of menstruation? For the primary outcome the responses from both these questions will be combined to calculate the proportion of girls with at least one-day absence during last period. For the secondary outcome responses from the question “In the last 30 days how many days of school did you miss because of menstruation?” will be used to ascertain the number of days missed in the last 30 days due to period.

### Secondary outcomes

#### Urinary tract infections symptoms

This secondary outcome was defined as proportion of girls reporting at least one urinary tract infection symptom over the 7 days preceding the survey. The questions used are listed in Table 2.

**Table 2:** Questions used in the survey to assess each outcome

Outcome	Questions in survey used to assess the outcome
School absenteeism (primary)	In the last 30 days how many days of school did you miss because of menstruation? or During your last period, how many days of school did you miss because of menstruation?
School absenteeism in last 30 days	In the last 30 days how many days of school did you miss because of menstruation?
Urinary tract infections symptoms	Over the 7 days preceding the survey, did you experience any of the following:
	1. Feeling of burning or discomfort when urinating,
	2. Have you had to wake up and pass urine more than usual
	3. Bad smell in urine
Reproductive tract infection symptoms	Over the 7 days preceding the survey, did you experience any of the following:
	1. Abnormal vaginal discharge (unusual texture and colour e.g., a milky vaginal discharge, more abundant than normal),
	2. Feeling of burning or itching in the genitalia
	3. Foul-smelling/fishy smell from genital area
Menstruation related wellbeing score while at school	1. How did you feel about continuing with your usual activities while menstruating at school?
	2. Do you feel comfortable/happy using the school toilets while menstruating?
	3. How did you feel about using your menstrual absorbent in the school this month?
	4. How confident/happy do you feel about participating in class during your period?
	5. How confident/happy do you feel when you are on your period as compared to when you are not
	6. Do you worry you do not have access to absorbent material when you are menstruating in school?
	7. Do you worry about what to do with the used absorbent material when you change in the school (or if you had to change in school)?
	8. Do you worry that the amount of water you have in the toilet at school is not enough to use the toilet or clean yourself when menstruating?
	9. Do you worry about staining your uniform at school when you are menstruating?
	10. Are you concerned people will know that you are menstruating, when you don’t go to pray while menstruating in school?
Social support	1. In general how did you feel about going to school during your last period?
	2. How did you feel about continuing with your usual activities while menstruating at home?
	3. How do you feel about talking with your mother/female caregiver about menstruation?
	4. How do you feel about talking with teachers about menstruation in school?
	5. How do you feel to talk about menstruation with other friends or school peers?
	6. Are you ever worried boys will tease you in school because you are menstruating? (options were not worried; worried; very worried)
	7. Do you feel your mother/care giver prepared you enough for menstruation? (options: well prepared; a bit prepared; not prepared at all)
Knowledge of menstruation and MH	1. When women are old do they menstruate?
	2. Is menstruation a disease?
	3. Do pregnant women menstruate?
	4. Does menstrual blood come from the stomach, where food is digested?
	5. Does menstrual blood came from the womb?
	6. Can a girl get pregnant before her first period?
	7. Must girls and women start their period on the same day every month?
	8. How long does a girl usually bleed for during her period?
	9. How long is the menstrual cycle?
Perceptions or disbelief of taboos towards menstruation and MH	1. Do disposable sanitary pads cause disease?
	2. If someone sees your used menstrual absorbent can it cause infertility?
	3. Is it acceptable to burn menstrual absorbents?
	4. Is it acceptable for a girl or woman to cook while menstruating?
	5. Is it acceptable for women to go out when they are menstruating?
	6. Is it acceptable for a girl to go to school while menstruating?
MH practices	1. How often did you change the absorbent material on one of the more heavy days of bleeding?
	2- In the past 3 months have you ever changed your menstrual absorbent in school?
	3. If you use reusable material, where is the material dried?

### Biochemical indicators of UTI

At the end of the survey, participants were requested to provide a mid-stream urine sample in a sterile specimen container. All specimen containers were labelled with participant’s study ID. The urine samples were analysed by the lead researcher (who had been trained to conduct these tests by the clinic staff at MRC Keneba) in the school setting, using urine dipstick analysis with the Combur 9-Test® strips [43]. This is a quick screening test for a UTI in children and adolescents, whose results are interpreted in the context of clinical feature suggestive of a UTI [44].

The guidance for urine collection that was given to the participants Additional file 6: Appendix S6.

Nitrites and leucocyte levels were measured using dipstick tests. A girl was considered to be UTI positive if she has a dipstick test positive for nitrites, or a dipstick test positive for leucocytes and at least one UTI symptom. All the girls that reported at least one UTI symptom or were positive for the nitrites indicator were referred to a clinic.

### Reproductive tract infection symptoms

This secondary outcome was defined as proportion of girls reporting at least one RTI symptom over the 7 days preceding the survey. The questions used are listed in Table 2.

### Menstruation related wellbeing score while at school

Prevalence of menstruation related wellbeing was measured at end-line among the adolescent girls using a new tool developed in this study. The items for the tool were developed through information gathered in the qualitative discussions during the formative study phase (between 2015 and 2017) and through information from enumerators and clinical staff. The questions were tested during the translation and pilot phase of the trial, and changed according to feedback. The tool was tested by enumerators, clinical staff, community volunteers, and school going adolescent girls. This tool consists of a set of 10 questions with binary response regarding menstruation related wellbeing while at school (happy, unhappy). The answers of these 10 questions will be reported individually as well as collapsed into a score using tetrachoric PCA. The questions used are listed in Table 2. In exploratory factor analysis we will look into the multidimensionality of the variable responses to explore the presence of relevant factor scores.

### Social support

A feeling tool developed in this study (that includes questions about comfort, confidence, and support from the mother and community when managing your menstruation) was used to assess the social support impact of the multipack intervention on participating girls. The tool was developed in the same way mentioned above. A set of 7 questions with three-level response regarding social support at home and school will be reported individually as well as collapsed into a score using polychoric PCA (happy, neither happy nor unhappy, or unhappy). Similar to the wellbeing score we will also do exploratory factor analysis for this score. The questions used are listed in Table 2.

### Knowledge of menstruation and MH

We evaluated knowledge using end-line questionnaires with closed questions, the outcome was defined as proportion of girls giving correct answers to knowledge based questions. A set of 9 questions with three level responses (yes, no, I don’t know) were used. The questions used are listed in Table 2.

### Perceptions or disbelief of taboos towards menstruation and MH

These were assessed through closed questions in the end-line survey. The outcome was defined as proportion of girls giving correct answers and disbelief of common taboos known in this context. A set of 6 questions with three level responses (yes, no, I don’t know) were used. The questions used are listed in Table 2.

### MH practices

These were assessed through closed questions in the end-line survey. The questions used are listed in Table 2.

### Cost and cost-effectiveness

We will conduct an economic evaluation to estimate the costs and cost-effectiveness of the MEGAMBO intervention. As a within-trial evaluation, the time horizon in the base case will be the length of follow-up, but we will also develop a decision analytic model to extrapolate costs and outcomes over a longer time horizon within which outcomes might be expected to be sustained for the targeted groups (e.g., 5 years). A combination of top-down and ingredients-based costing approaches will be used to generate cost estimates for the whole intervention and for each component. All costs will be estimated from disaggregated social perspective (both provider and societal) and financial and economic costs will be calculated for all inputs. The results will provide the costs of setting up and running the intervention package, describe the distribution of costs across 4 intervention components, the unit cost per student reached and the cost of delivering all activities in intervention schools. Since no intervention is undertaken in control schools, these costs are by definition incremental over control. Primary and selected secondary outcome measures will be used for the cost-effectiveness analysis of the intervention relative to usual practice (represented by the control schools). For example, cost per percentage point change in the primary outcome, and cost per school day gained. If applicable, we will estimate the cost per disability-adjusted life year averted. Since outcomes are across many domains and cannot be aggregated, we will present overall cost-effectiveness results as a cost-consequences analysis.

## Statistical analyses

For binary outcome variables we will use binomial regression with the identity link to produce prevalence differences. Clustering at school level will be adjusted for by using GEE models with robust standard errors. For count variables (number of days missed) we will use Poisson regression. Over-dispersion at cluster level (school) will be adjusted for by using GEE. Effect estimates will be expressed as the rate difference (additive model). The strata used for randomisation will be included as an indicator variable. In a secondary analysis we will adjust the model for all balance variables used for the restricted randomisation.

Planned subgroup analyses will be done for school type (English vs Arabic), degree of urbanization (dichotomised, the cut-off will be determined prior to rolling out the full trial) and school size (< 150 girls vs. ≥ 150 girls enrolled).

### Sensitivity analysis

Because of COVID-19 restrictions, data collection in five schools could only be done 7 months after the other schools had been completed. A longer gap between intervention and outcome assessment could have influenced the way girls responded to the questionnaire, affect recall of training. There is a possibility that over this time period, girls became more confident and knowledgeable in managing their menstruation through experience, or girls that were having more challenges may have dropped out during this time. We will conduct a sensitivity analysis by exploring the robustness of the main effect estimates to excluding these 5 schools from the analysis, with or without adjustment for covariates in case the exclusion leads to relevant imbalances across arms. We have collected information on number of girls that have dropped out per school to assess level of dropout rates over the time period.

## Discussion

This RCT study is one of the first to evaluate the effect of a multicomponent intervention to improve MH and school attendance among school girls on a large scale. The findings from this trial could contribute to highlight methods to increase school attendance and the retention of adolescent girls in school for longer and could have benefits across multiple areas such as health, education and development [3, 11, 12, 23, 24, 45, 46].

Some study considerations may be especially important in designing evaluations of multicomponent MH interventions in order to deal with the complexity of the subject. First, process evaluation—documenting the manner in which each component of the intervention is actually implemented rather than intended—is essential to put outcomes into perspective. This information will also inform and strengthen the development of programmatic materials for implementation, should the trial show positive outcomes. Secondly, the restricted randomisation design provides rigorous means of improving balance of potential confounders. Although there is risk of response bias for reported outcomes, it is unlikely to be different by intervention status since the implementation team was different to the evaluation team. It is also unlikely that the evaluation team was aware of the intervention status, as they were kept blinded to the components of the intervention. Baseline outcome data was not collected, so as to reduce risk of introducing bias [47].

The study included girls, boys, mothers, and male members of the community, as sustainable change in menstrual experiences depends on addressing social taboos and stigmas among all members of the community. This is the first trial that assessed the impact of a MH intervention on adolescent girls’ urinary tract infections by using rapid diagnostic dipstick techniques in the field. It also used innovative tools, developed specifically for the purpose of the trial, to measure menstruation related wellbeing and social support, which will hopefully provide better indication of what areas need to be tackled more and what the intervention packages have improved. Since the development of these tools, there has been great movement in the field and other tools have been developed, e.g., the MPNS-36 [48] and the MENISCUS stress tool (unpublished).

Efforts have been made from the beginning of the study to involve Gambian education government departments, Gender Unit department and different relevant NGOs working on MH, adolescent and gender issues in The Gambia in order to strengthen the design of the intervention that could be integrated in the future into existing programmes.

Some study limitations are, there are no gold standard measurements of school attendance especially in this context, therefore the primary outcome data was collected through self-reported recall over a 30 day period, however this may be subject to respondent or recall bias.

Due to limited time and resources it was not possible to have refresher sessions for the different components of the intervention, however during each interaction with the different groups, messages of sharing the information learnt was stressed multiple times, we hope this combined with the multicomponent design can result in people hearing and integrating the message multiple times.

COVID-19 affected completion of end-line data collection, the schools where data was collected after the long break could differ from those that were collected before the break. Potential reasons for the difference is the longer period could cause more recall bias, and the girls could have forgotten the messages from the training, the school environment could be different, and the WASH facilities be improved due to new government COVID-19 guidelines. The pandemic also led to fear and distrust of outsiders and medical institutions by some members of the public, which resulted in the drop out of one school, as they did not want anyone from the Medical Research Council, Unit The Gambia (MRCG) to interact with their students. This can cause an imbalance and reduction of power of the study. However, all this will be adjusted for during analysis. The school that dropped out was a relatively small school, so should not affect sample size drastically.

## Conclusion

The intervention included girls, boys, mothers and community male members, as sustainable changes in menstrual management depend on addressing social taboos and stigmas among the full community. We discussed a number of problems measuring school attendance and having verification systems, this may be typical in school based studies that measure school attendance. Nevertheless, it is expected that these problems are present in both control and intervention schools, so will not bias measurements of effectiveness of the interventions on the outcomes. The process evaluation data collected should shed light on what aspects of the study can be improved for future roll out.

Results from the trial (expected August 2022) may help inform policy. Results will elude on effectiveness of MH interventions, explore validity of measurement approaches, describe fidelity of implementation and potential challenges or successes. A positive result may encourage policy makers to increase their commitment to improve operation and maintenance of school WASH facilities and include more information on menstruation and puberty into the curriculum, as well as considering campaigns in communities to reduce the taboos and stigmas linked to menstruation, especially targeted at the men as they have the least information on the matter. A negative result will provide opportunities to improve MH interventions in the future.

## Supplementary Information

**Additional file 1: Appendix S1.** Consent forms for participants over 18 years.

**Additional file 2: Appendix S2.** Consent/Assent forms for participants under 18 years.

**Additional file 3: Appendix S3.** School WASH facilities spot check.

**Additional file 4: Appendix S4.** Theory of change (TOC).

**Additional file 5: Appendix S5.** Main study questionnaire.

**Additional file 6: Appendix S6.** Urine collection protocol.

## Data Availability

Not applicable: our manuscript does not contain any data or related findings. Additional material of tools used are provided.

## Abbreviations

MH: Menstrual health
WASH: Water sanitation and hygiene
UTI: Urinary tract infects
RTI: Reproductive tract infects
MoBSE: Ministry of Basic and Secondary Education
LBS: Lowe basic school
BCS: Basic cycle school
UBS: Upper basic school
SSS: Senior secondary school
LRR: Lower river region
NBR: North Bank Region
NSGA: Nova Scotia Gambia Association
TOR: Terms of reference
IDI: In depth interviews
FGD: Focus group discussions
MRCG: Medical Research Council, Unit The Gambia
