# Supplementary material for: Puberty health intervention to improve menstrual health and school attendance among adolescent girls in The Gambia: study methodology of a cluster-randomised controlled trial in rural Gambia (MEGAMBO TRIAL)
Source: Emerg Themes Epidemiol. 2022 Jul 16;19:6. doi: 10.1186/s12982-022-00114-x (PMC9287699; doi:10.1186/s12982-022-00114-x)
Supplement: Supplementary file 1 — Additional file 1: Appendix S1. Consent forms for participants over 18 years. [file 12982_2022_114_MOESM1_ESM.docx]

**PARTICIPANT INFORMATION SHEET**

| Version | 1.0 | Date | 05/10/2018 |
| --- | --- | --- | --- |

Study Title: Puberty health intervention to improve menstrual health and School attendance among adolescent girls in Gambia.

| SCC: | 1633 | Protocol: |  |
| --- | --- | --- | --- |

Sponsor & Funder: London School of Hygiene and Tropical Medicine and MRC The Gambia Unit

## What is informed consent?

You are invited to take part in a research study. Participating in a research study is not the same as getting regular medical care. The purpose of normal medical care is to improve one’s health. The purpose of a research study is to gather information that may be useful in the future for the whole population. It is your decision to take part and you can stop at any time without giving any reason. Please take time to read the following information or get the information explained to you in your language. Listen carefully. You can ask questions if there is anything that you do not understand. Ask for it to be explained until you are satisfied. You may also wish to speak your spouse, family members or others before deciding to take part in the study.If you decide to join the study, you will need to sign or thumbprint a consent form saying you agree to be in the study. You will receive a copy of the consent form.

## Why is this study being done?

The London School of Hygiene and Tropical Medicine (LSHTM) and MRC Gambia are conducting research to find out what students know about changes that occur during puberty and the differences between boys and girls. We are also interested in the wellbeing of girls in school. The results of the study will be made available to your community and the regional education office.

## What does this study involve?

We will carry out various training sessions, and conduct group discussions with different members of the community. We will also be asking questions to gather information related to menstruation, school life and some other health outcomes.

To assess for urine infections, the female students will be asked a series of questions about their health and also will be asked to donate a urine sample, where infection will be measured. If their response to any of the questions and/or the urine test results suggest a urine infection then, they will be referred to the MRC Keneba clinic for further assessment and treatment.

If we find out that you are sick and cannot join the study, you will receive the care routinely available in The Gambia. You may be treated at the study site and if necessary you will be referred to a health facility that can manage the condition better.

If the research study needs to be stopped for any reason, we will tell you and you will have normal medical care if you need it.

## What will happen to the samples taken in this study?

Urine samples will be tested in the field, and then safely discarded at MRC.

## What harm or discomfort can you expect in the study?

Some may find it difficult to talk to us or may have trouble from other members of the community for talking to us. However, we will spend a lot of time with you and the community explaining the nature of the study to community leaders prior to starting the interviews, to prevent any misunderstandings.

## What benefits can you expect in the study?

You will better understand changes that occur during puberty by the end of the study. If there are any misconceptions or myths around the topic, your wellbeing can potentially be improved through honest discussions. It may also facilitate early assessment and management of urine infections.

## Will you be compensated for participating in the study?

You will not get paid by the study.

## What happens if you refuse to participate in the study or change your mind later?

You are free to join the study or not and you are free to stop being in the study any time without giving a reason. You will still get the normal medical care.

If you do not want to continue in the study, we will use only the samples and information already collected from you.

If we find new information during the study that may change if you can still be in the study, we will inform you as soon as possible.

## How will personal records remain confidential and who will have access to it?

All information that is collected about you in the study will be kept strictly confidential. Your personal information will only be seen by the study team members, the sponsor and if necessary the Ethics Committee and Government authorities.

## Who should you contact if you have questions?

If you have any questions or are worried you can call Mrs Fatou Sosseh on 7008530 or Miss Vishna Shah on 2881480 and you can always call the personal numbers of the MRC workers given to you.

Please feel free to ask any question you might have about the study.

## Who has reviewed this study?

This study has been checked by scientists at the Medical Research Council and by the Gambia Government/MRC Joint Ethics Committee. The Ethics Committee protects your rights and wellbeing, and has given permission for it to take place.

**Consent Form**

Participant Identification Number: |__|__|__|__|__|__|__|__|__|__|__|__|

(Printed name of participant)

I have read the written information **OR**

I have had the information explained to me by study personnel in a language that I understand,

and I

- confirm that my choice to participate is entirely voluntarily,
- confirm that I have had the opportunity to ask questions about this study and I am happy with the answers that have been provided,
- understand that I allow access to the information about me by the persons described in the information sheet,
- had enough time to think about whether I want to take part in this study,
- agree to take part in this study.

*Tick as appropriate*

| I agree for my samples to be shipped outside of The Gambia | Yes  No | | | |
| --- | --- | --- | --- | --- |
| I agree to further research on my samples including genetic testing | | Yes | No |  |

| Participant’s signature/ thumbprint* |  |  |  |  |
| --- | --- | --- | --- | --- |
|  |  |  | Date (dd/mmm/yyyy) Time (24hr) | |
|  |  |  |  | |
| Printed name of witness* |  | | | |
| Printed name of person obtaining consent |  | | | |
| **I attest that I have explained the study information accurately in** ____________________**__ to, and was understood to the best of my knowledge by, the participant. He/she has freely given consent to participate ***in the presence of the above named witness (where applicable).** | | | | |
| Signature of person obtaining consent |  |  |  | |
|  |  |  | Date (dd/mmm/yyyy) Time (24hr) | |
| ** Only required if the participant is unable to read or write.* | | | | |
