# Supplementary material for: Puberty health intervention to improve menstrual health and school attendance among adolescent girls in The Gambia: study methodology of a cluster-randomised controlled trial in rural Gambia (MEGAMBO TRIAL)
Source: Emerg Themes Epidemiol. 2022 Jul 16;19:6. doi: 10.1186/s12982-022-00114-x (PMC9287699; doi:10.1186/s12982-022-00114-x)
Supplement: Supplementary file 3 — Additional file 3: Appendix S3. School WASH facilities spot check. [file 12982_2022_114_MOESM3_ESM.pdf]

Main Study Wash Spotcheck

Study ID: ____________________

SECTION 1: School Facilities Observations

1.1 - Observers name	◯ Vishna Shah<br>◯ Isatou Jammeh<br>◯ Tida Samateh
1.2 - Date	____________________
1.3 - Visit:	◯ Start<br>◯ Middle<br>◯ End
1.4 - Total number of girls	____________________
1.5 - Total number of boys	____________________
1.6 - Total number of female teachers	____________________
1.7 - Total number of male teachers	____________________
1.8 - Does the school have any sanitary pads supply?	◯ Yes<br>◯ No
1.9 - Number of disposal sanitary pads at visit	____________________
1.10 - Date of last sanitary pad delivery	____________________

The date should be before the date of observation, not after, usually max 2 years before the date of observation.

1.11 - Number of disposal sanitary pads supplied by government	____________________
1.12 - Number of disposal sanitary pads left when you got the last delivery	____________________

SECTION 2: Sanitation Observations

2.1 - Are there toilets/latrines at the school?
◯ Yes
◯ No

2.2 - Type of latrines in school
☐ Ventilated improved pit latrine (VIP)
☐ Flush/pour flush latrine
☐ Pit latrine with slab
☐ Pit latrine without slab

2.3 - How many blocks they are?

a) Total ______________________

b) Girls ______________________

c) Boys ______________________

d) Mix ______________________

e) Teachers ______________________

2.4 - Where are the latrines located?
◯ Near the classrooms
◯ A private space away from the classroom

2.5 - Are girls and boys blocks adjacent to each other?
◯ Yes
◯ No

SECTION 3:
3.1 - Fill the table below:

a) Total number of cubicles per block: ______________________

b) Locked ______________________

c) Functional ______________________

e) Light ______________________

d) Clean ______________________

f) Full door ______________________

g) Partial door ______________________

h) Lock inside ______________________

i) Lockable ______________________

j) Roof ______________________

k) Walls high ______________________

l) Bins inside ______________________

m) Water Inside ______________________

a) Total number of cubicles per block: ______________________

b) Locked ______________________

c) Functional ______________________

d) Clean ______________________

e) Light ______________________

f) Full door ______________________

g) Partial door ______________________

h) Lock inside ______________________

i) Lockable ______________________

j) Roof ______________________

k) Walls high ______________________

l) Bins inside ______________________

m) Water Inside ______________________

a) Total number of cubicles per block: ______________________

b) Locked ______________________

c) Functional ______________________

d) Clean ______________________

e) Light ______________________

f) Full door ______________________

g) Partial door ______________________

h) Lock inside ______________________

i) Lockable ______________________

j) Roof ______________________

k) Walls high ______________________

l) Bins inside ______________________

m) Water Inside ______________________

a) Total number of cubicles per block: ______________________

b) Locked ______________________

c) Functional ______________________

d) Clean ______________________

e) Light ______________________________

f) Full door ______________________________

g) Partial door ______________________________

h) Lock inside ______________________________

i) Lockable ______________________________

j) Roof ______________________________

k) Walls high ______________________________

l) Bins inside ______________________________

m) Water Inside ______________________________

a) Total number of cubicles per block: ______________________________

b) Locked ______________________________

c) Functional ______________________________

d) Clean ______________________________

e) Light ______________________________

f) Full door ______________________________

g) Partial door ______________________________

h) Lock inside ______________________________

i) Lockable ______________________________

j) Roof ________________________________

k) Walls high ________________________________

l) Bins inside ________________________________

m) Water Inside ________________________________

3.2 - Enter number of latrines/toilets for each category

SECTION 4: Menstrual Absorbent Disposal

4.1 - At the time of the visit, did you see a bin for disposing used menstrual blood absorbents?
- ◯ Yes
- ◯ No

4.2 - If yes, where were they present?
- ◯ Inside all cubicles
- ◯ Inside some cublicles
- ◯ Outside the block

4.3 - At the time of the visit, did you see a pit for burning used menstrual blood absorbents?
- ◯ Yes
- ◯ No

SECTION 5: Hand-Washing Observations

5.1 - Does the school have hand-washing facilities?
- ◯ Yes
- ◯ No

5.2 - What kind of hand-washing facilities does the school have?
- ☐ Running water from a piped system or tank
- ☐ Hand-poured water system (bucket or ladle)
- ☐ Basin/bucket (hand-washing done in the water and is not running or poured)
- ☐ Other

5.3 - Specify other hand washing facility ________________________________

5.4 - How many hand washing facilities are located outside (but within school grounds)? ________________________________

5.5 - How many hand washing facilities are located inside/close to the classrooms? ________________________________

5.6 - Is there handwashing facilities close to the toilet block?
- ◯ Yes
- ◯ No

5.7 - Do you find soap on the hand-washing facilities?
- ◯ Almost always
- ◯ In some of them
- ◯ No soap at all

SECTION 6: Water Access Observations

6.1 - Is there water available in the school?

◯ Yes
◯ No

6.2 - What are the sources of water available at school?

☐ Piped water into school building
☐ Piped water to schoolyard
☐ Surface water (river, lake, steam)
☐ Cart with small tank/water tank/jerrican
☐ Public tap/standpipe
☐ Tube well/borehole
☐ Protected well/spring
☐ Unprotected well/spring
☐ Rainwater collection
☐ Other source, specify
(Select all that apply)

6.3 - Specify other water availability

____

6.4 - What is the main water source available at the school?

◯ Piped water into school building
◯ Piped water to schoolyard
◯ Surface water (river, lake, stream)
◯ Cart with small tank/water tank
◯ Public tap/standpipe
◯ Tube well/borehole
◯ Protected well/spring
◯ Unprotected well/spring
◯ Rainwater collection
◯ Other

6.5 - Specify other main water source

____

6.6 - Is there always water available at the main source of water

◯ All the time
◯ Few hours in the morning
◯ Few hours in the afternoon
◯ Few days per week
◯ Not available
◯ Other

6.7 - Specify other times water is available at the main source

____

6.8 - At the time of the visit, was the main water source functional?

◯ Yes
◯ No

6.9 - Was water available around the toilets?

◯ Yes
◯ No

6.10 - Why is there no water around the toilets?

____

6.11 - What type of water was available around the toilet?

☐ Piped water
☐ Bucket stores
☐ Tippy tap
(mark all that applies)

6.12 - Where was the bucket provided placed?	◯ Near the toilet<br>◯ Near the kitchen<br>◯ Near the classrooms<br>◯ Near the water source<br>◯ Not seen<br>◯ Other
6.13 - Was there water in the bucket?	◯ Yes<br>◯ No
6.14 - General comments	____

____
