# Supplementary figures and images for: Puberty health intervention to improve menstrual health and school attendance among adolescent girls in The Gambia: study methodology of a cluster-randomised controlled trial in rural Gambia (MEGAMBO TRIAL)
Source: Emerg Themes Epidemiol. 2022 Jul 16;19:6. doi: 10.1186/s12982-022-00114-x (PMC9287699; doi:10.1186/s12982-022-00114-x)

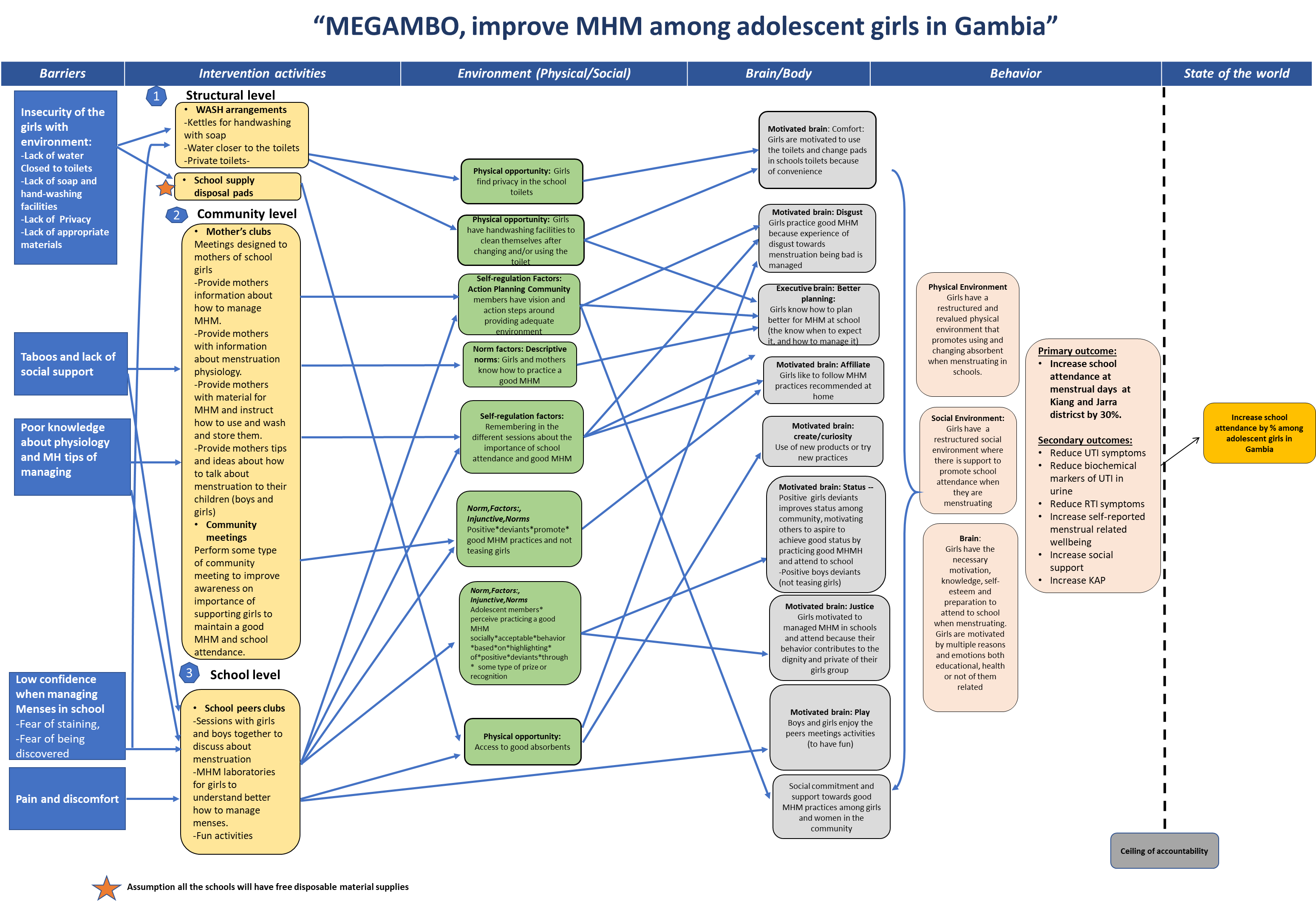


**Appendix S4: Theory of change**

Supplement: Supplementary file 4 — Additional file 4: Appendix S4. Theory of change (TOC). [file 12982_2022_114_MOESM4_ESM.docx]

Appendix S6. Urine collection protocol


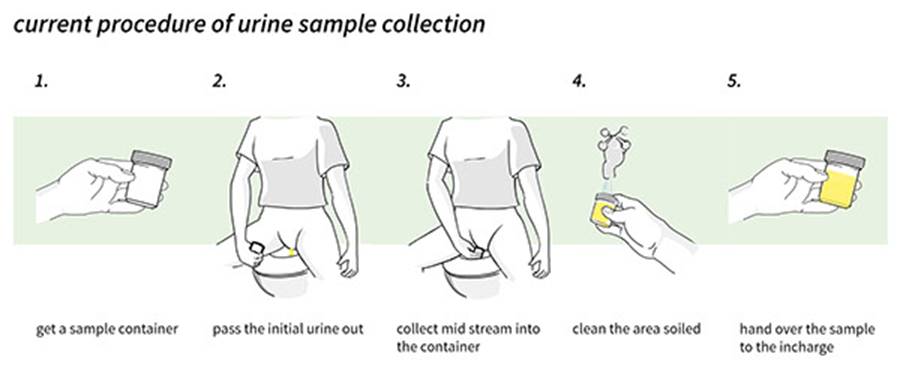

Supplement: Supplementary file 6 — Additional file 6: Appendix S6. Urine collection protocol. [file 12982_2022_114_MOESM6_ESM.docx]
