# Supplementary material for: Puberty health intervention to improve menstrual health and school attendance among adolescent girls in The Gambia: study methodology of a cluster-randomised controlled trial in rural Gambia (MEGAMBO TRIAL)
Source: Emerg Themes Epidemiol. 2022 Jul 16;19:6. doi: 10.1186/s12982-022-00114-x (PMC9287699; doi:10.1186/s12982-022-00114-x)
Supplement: Supplementary file 5 — Additional file 5: Appendix S5. Main study questionnaire. [file 12982_2022_114_MOESM5_ESM.pdf]

Main Study Questionnaire

Study ID:	__________________________________
1.2 - Date:	__________________________________
1.3 - Religion:	◯ Muslim<br>◯ Christian<br>◯ Other
1.4 - Specify other religion:	__________________________________
1.5 - How do you reach school?	◯ Walking<br>◯ Cycling<br>◯ Vehicle<br>◯ Other
1.6 - Specify other means:	__________________________________
1.7 - What level of education has your mother received?	◯ No formal education<br>◯ Arabic<br>◯ Some Primary<br>◯ Completed Primary<br>◯ Some Secondary<br>◯ Completed Secondary<br>◯ Further education (university, etc.)<br>◯ I Don't Know
1.8 - What level of education has your father received?	◯ No formal education<br>◯ Arabic<br>◯ Some Primary<br>◯ Completed Primary<br>◯ Some Secondary<br>◯ Completed Secondary<br>◯ Further education (university, etc.)<br>◯ I Don't Know
1.9 - What are the walls of your household made of?	◯ Mud<br>◯ Cement<br>◯ Other
1.10 - Specify other wall:	__________________________________
1.11 - What are the floors of your household made of?	◯ Mud<br>◯ Cement<br>◯ Tile<br>◯ Other
1.12 - Specify other floors:	__________________________________

1.13 - Do you have or own any of the following items?

- ☐ Sofa
- ☐ Radio
- ☐ Television
- ☐ Refrigerator
- ☐ Gas cooker
- ☐ Mobile
- ☐ Electricity/Solar
- ☐ Bicycle
- ☐ Motorbike
- ☐ Car or Truck
- ☐ Animal cart
- ☐ Boat without motor
- ☐ Boat with motor

(Select all that apply)

1.14 - Over the past 6 months, what is the main source of water for the members of your household?

- ◯ Unprotected well (polliyo)
- ◯ Protected well
- ◯ Community hand pump
- ◯ Community standpipe
- ◯ Household water
- ◯ Other

1.15 - If Other, please specify:

____

1.16 - How long does it take to go there, get water, and come back?

- ◯ Less than 30 minutes
- ◯ More than 30 minutes

1.17 - What is the main toilet facilities members of your household usually use?

- ◯ Pit latrine without a slab
- ◯ Pit latrine with a slab
- ◯ Pour flush/flush toilet
- ◯ Other

1.18 - If 'Other', please specify:

____

1.19 - How many bathrooms you have in your house?

- ◯ No bathroom
- ◯ Separate for men and women
- ◯ Bathroom shared by women and men

SECTION 2. Missing School

2.1 - In the last 30 days how many days of school did you miss?

2.2 - In the last 30 days how many days of school did you miss?

- ◯ 0
- ◯ 1
- ◯ 2
- ◯ 3
- ◯ 4
- ◯ 5
- ◯ 6
- ◯ 7
- ◯ 8
- ◯ 9
- ◯ 10
- ◯ Other

2.3 - Specify other number of days missed:

____

2.4 - In the last 30 days how many days of school did you miss because of domestic duties?

- ◯ 0
- ◯ 1
- ◯ 2
- ◯ 3
- ◯ 4
- ◯ 5
- ◯ 6
- ◯ 7
- ◯ 8
- ◯ 9
- ◯ 10
- ◯ Other

2.5 - Specify other number of days missed:

____

2.6 - In the last 30 days how many days of school did you miss because of fever or diarrhea?

- ◯ 0
- ◯ 1
- ◯ 2
- ◯ 3
- ◯ 4
- ◯ 5
- ◯ 6
- ◯ 7
- ◯ 8
- ◯ 9
- ◯ 10
- ◯ Other

2.7 - Specify other number of days missed

____

2.8 - In the last 30 days how many days of school did you miss because of menstruation?

- ◯ 0
- ◯ 1
- ◯ 2
- ◯ 3
- ◯ 4
- ◯ 5
- ◯ 6
- ◯ 7
- ◯ 8
- ◯ 9
- ◯ 10
- ◯ Other

2.9 - Specify other number of days missed

____

2.10 - In the last 30 days how many days of school did you miss because of menstrual pain?

- ◯ 0
- ◯ 1
- ◯ 2
- ◯ 3
- ◯ 4
- ◯ 5
- ◯ 6
- ◯ 7
- ◯ 8
- ◯ 9
- ◯ 10
- ◯ Other

2.11 - Specify other number of days missed	__________________
2.12 - Did you menstruate in the last 30 days?	◯ Yes<br>◯ No
2.13 During your last period how many days of school did you miss?	◯ 0<br>◯ 1<br>◯ 2<br>◯ 3<br>◯ 4<br>◯ 5<br>◯ 6<br>◯ 7<br>◯ 8<br>◯ 9<br>◯ 10<br>◯ Other
2.14 - Specify other number of days missed	__________________
2.15 - During your last period how many days of school did you miss because of menstrual pain?	◯ 0<br>◯ 1<br>◯ 2<br>◯ 3<br>◯ 4<br>◯ 5<br>◯ 6<br>◯ 7<br>◯ 8<br>◯ 9<br>◯ 10<br>◯ Other
2.16 - Specify other number of days missed	__________________
2.17 - Average level of pain experienced during your period?	◯ No pain<br>◯ Mild pain<br>◯ Extreme pain
2.18 - Has menstrual ever made you miss school?	◯ Yes<br>◯ No
2.19 - Has menstrual pain ever made you miss school?	◯ Yes<br>◯ No

SECTION 3. Knowledge and Attitudes about Menstruation.

3.1 - At what age did you have your first menstrual period? ________________________

3.2 - Did you learn about menstruation before or after menarche?
- ◯ Before
- ◯ After

3.3 - What age did you learn about menstruation? ________________________

(**I want open text or have not learnt as yet, is that possible?)

3.4 - Who do you talk to about menstruation the most?
- ◯ Grandmother
- ◯ Mother
- ◯ Sister
- ◯ Father
- ◯ Teacher
- ◯ Peers
- ◯ Other

3.5 - If Other, please specify: ________________________

3.6 - When you were told about menstruation, what did you talk about?
- ☐ How to be clean
- ☐ What absorbent material to use
- ☐ How to use/wash/dry/dispose the absorbent material
- ☐ How to manage pain
- ☐ What menstruation is
- ☐ How to know when you will expect your next period
- ☐ To abstain from sex/avoid contact with boys
- ☐ Have not talked about it
- ☐ Other

(Select all that apply)

3.7 - If Other, please specify: ________________________

	No	Yes	I Don't Know
3.8 - When women are very old, do they menstruate?	◯	◯	◯
3.9 - Is menstruation a disease?	◯	◯	◯
3.10 - Do pregnant women menstruate?	◯	◯	◯
3.11 - Does menstrual blood come from the stomach where the food is digested?	◯	◯	◯
3.12 - Does menstrual blood come from the womb?	◯	◯	◯
3.13 - Do disposable sanitary pads cause disease?	◯	◯	◯
3.14 - Is it safe for a girl to dry her reusable material under the mattress?	◯	◯	◯
3.15 - Can a girl get pregnant before having her first period?	◯	◯	◯
3.16 - If someone sees/gets your used menstrual absorbent can it cause infertility?	◯	◯	◯
3.17 - Must girls and women start their period on the same day every month?	◯	◯	◯
3.18 - Is it acceptable to burn menstrual absorbents?	◯	◯	◯
3.19 - Is it acceptable for a girl or woman to cook while menstruating?	◯	◯	◯
3.20 - Is it acceptable for women to go out when they are menstruating?	◯	◯	◯
3.21 - Is it acceptable for a girl to go to school while menstruating?	◯	◯	◯

3.22 - How long does a girl usually bleed for during her period?

- ◯ Between 2 to 7 days
- ◯ Exactly 28 days
- ◯ Exactly 5 days
- ◯ 1 day
- ◯ Exactly 31 days
- ◯ Between 21-45 days
- ◯ I don't know

3.23 - How long is the menstrual cycle (i.e. the number of days between periods?)

- ◯ Between 2 to 7 days
- ◯ Exactly 28 days
- ◯ Exactly 5 days
- ◯ 1 day
- ◯ Exactly 31 days
- ◯ Between 21-45 days
- ◯ I don't know

SECTION 4. Menstrual Hygiene Management Practices

4.1 - Now we are going to ask some questions regarding your normal habits of dealing with menstruation.

4.2 - Which of these products used to absorb menstrual blood have you heard of?	☐ Reusable local materials like cloth or towel<br>☐ Commercially made reusable cloth/pads<br>☐ Disposable sanitary pad<br>☐ Tampon<br>☐ Menstrual cup<br>(Select all that apply)
4.3 - What absorbent material did you usually use during your last period at home?	◯ Reusable local materials like cloth or towel<br>◯ Commercially made reusable cloth/pads<br>◯ Disposable sanitary pad<br>◯ Both disposal and reusable<br>◯ None<br>◯ Other
4.4 - Specify other absorbent material:	______________________________
4.5 - What absorbent material did you usually use during your last period at school?	◯ Reusable local materials like cloth or towel<br>◯ Commercially made reusable cloth/pads<br>◯ Disposable sanitary pad<br>◯ Both disposal and reusable<br>◯ None<br>◯ Other
4.6 - Specify other absorbent material:	______________________________
4.7 - Which absorbent do you prefer using?	◯ Reusable local materials like cloth or towel<br>◯ Commercially made reusable cloth/pads<br>◯ Disposable sanitary pad
4.8 - How often would you change the absorbent material on one of the more heavy days of bleeding?	◯ Once a day<br>◯ Twice a day<br>◯ Three times a day or more
4.9 - Does your school give girls a supply of pads?	◯ Yes<br>◯ No
4.10 - Have you gone to request any pads this year?	◯ Yes<br>◯ No
4.11 - How many pads were you given the last time you went to collect some?	______________________________
4.12 - In the past 3 months have you ever changed your menstrual absorbent in school?	◯ Yes<br>◯ No
4.13 - What do you normally do with your used menstrual absorbent that you have changed at school?	◯ Throw it in the school toilet<br>◯ Bring it home<br>◯ Throw it away in any other place

4.14 - If you use reusable material, where is the material dried?

- ◯ Under the mattress
- ◯ Dry it inside the house or a room
- ◯ In a bathroom or toilet with a roof
- ◯ In a bathroom or toilet without a roof
- ◯ Dry it in the sun or open space
- ◯ Does not use reusable menstrual absorbent
- ◯ Other

(Please select the main one)

4.15 - Specify other drying method: __________________________________

SECTION 5. Menstrual Stress When in School

	Unhappy	Happy
5.1 - How did you feel to continue with your usual activities while menstruating at school?	◯	◯
5.2 - Do you feel comfortable/happy using the school toilets while menstruating?	◯	◯
5.3 - How did you feel about using your menstrual absorbent in the school this month?	◯	◯
5.4 - How confident/happy do you feel about participating in class during your period?	◯	◯
5.5 - How confident/happy do you feel when you are on your period as compared to when you are not -	◯	◯

	Yes	No
5.6 - Do you worry you do not have access to absorbent material when you are menstruating in school?	◯	◯
5.7 - Do you worry about what to do with the used absorbent material when you change in the school (or if you had to change in school)?	◯	◯
5.8 - Do you worry that the amount of water you have in the toilet at school, is not enough to use the toilet or clean yourself when menstruating?	◯	◯
5.9 - Do you worry about staining your uniform at school when you are menstruating?	◯	◯
5.10 - Are you concerned people will know that you are menstruating, when you don't go to pray while menstruating in school?	◯	◯

SECTION 6. Social (Community and School) Support

	UnHappy	Neither Happy Nor Unhappy	Happy
6.1 - In general how did you feel about going to school during your last period?	◯	◯	◯
6.2 - How did you feel to continue with your usual activities while menstruating at home?	◯	◯	◯
6.3 - How do you feel to talk with your mother/female care giver about menstruation?	◯	◯	◯
6.4 - How do you feel to talk with teachers about menstruation in school?	◯	◯	◯
6.5 - How do you feel to talk about menstruation with other friends or school peers?	◯	◯	◯

6.6 - Are you ever worried boys will tease you in school because you are menstruating?

◯ Very Worried
◯ Worried
◯ Not Worried

6.7 - Do you feel your mother/care giver prepared you enough for menstruation?

◯ Not prepared at all
◯ A bit prepared
◯ Well prepared

6.8 - In the past 6 months who have you heard about menstruation from?

- ☐ Grandmother
- ☐ Mother
- ☐ Sister
- ☐ Father
- ☐ Teacher
- ☐ Peer health councilors
- ☐ External trainers
- ☐ No-one
- ☐ Other

6.9 - Specify other: ________________________

SECTION 7. Symptoms of Urinary Tract Infections

7.1 - Did you have any of the following symptoms in the last Week?

	Yes	No
7.2 - Feeling of burning or discomfort when urinating -	○	○
7.3 - Have you had to wake up and pass urine more than usual?	○	○
7.4 - Have you noticed your urine is smelling bad?	○	○

SECTION 8. Symptoms of Reproductive Tract Infections

8.1 - Did you have any of the following symptoms in the last Week?

	Yes	No
8.2 - Abnormal vaginal discharge (unusual texture and color e.g. a milky vaginal discharge, more abundant than normally)	○	○
8.3 - Feeling of burning or itching in the genitalia.	○	○
8.4 - Foul-smelling/fishy smell from genital area?	○	○

SECTION 9.Intervention Exposure

9.1 - At School did you have a session on menstruation last term?

- ○ Yes
- ○ No

9.2 - Who did you have the session with?

- ☐ Teacher
- ☐ Peer health Coordinators
- ☐ An outsider to the school
- ☐ other

(Select all that apply)

9.3 - Specify other ________________________

9.4 - Did you attend any of the sessions?
- ◯ Yes
- ◯ No

9.5 - Did you see any posters about menstruation around the school?
- ◯ Yes
- ◯ No

9.6 - Did you receive any material with information about menstrual tips?
- ◯ Yes
- ◯ No

9.7 - Did you learn how to track your menstruation cycle?
- ◯ Yes
- ◯ No

9.8 - Do you know if your parents had any talks about menstruation?
- ◯ Yes
- ◯ No

9.9 - Which parent attended the talks?
- ☐ Mother
- ☐ Father

Section 10. COVID-19

10.1 - During the past 30 days, how stressful have the COVID-related restrictions on living home been for you?
- ◯ Not at all stressful
- ◯ Somewhat stressful
- ◯ Very stressful

10.2 - Since the school closures due to COVID-19, has it been more difficult to obtain the type of sanitary product you normally use?
- ◯ Yes
- ◯ No

10.3 - If yes, specify the difficulties you faced.

____

10.4 - Have you observed any of the following changes in the school since they re-opened?
- ☐ More toilets functioning
- ☐ Less toilets functioning
- ☐ More hand-washing stations
- ☐ Less hand-washing stations
- ☐ More soap available
- ☐ Less soap available
- ☐ More Kettles available
- ☐ Less kettles available
- ☐ Posters
- ☐ Other changes
- ☐ None of the above

(Select all that applies and specify if 'Other Changes' happened)

10.5 - Specify if other changes happened.

____

10.6 - When schools reopened, were you happy to return to school?
- ☐ Yes
- ☐ No
- ☐ Indifferent

10.7 - Since you school reopened are you more likely or less likely to attend school?
- ◯ More likely
- ◯ Less likely
- ◯ No change

10.8 - Have you had any concerns attending school since they reopened?

◯ Yes
◯ No

10.9 - If yes, explain

____

Username

____

Main Study Urine Test

Study ID: ______________________

Urine Collection

1 - Sample tested by:
- ◯ Vishna Shah
- ◯ Isatou Jammeh

2 - Date ______________________

3 - Was urine sample given?
- ◯ Yes
- ◯ No

4 - If no, why was a sample not given? ______________________

Urine dip stick test results

5 - Leukocytes:
- ◯ +
- ◯ ++
- ◯ +++
- ◯ Negative

6 - Nitrites:
- ◯ Positive
- ◯ Negative

7 - Blood:
- ◯ Positive
- ◯ Negative

8 - Overall dipstick test:
- ◯ Positive
- ◯ Negative

____
